# T1-weighted MRI texture analysis in amyotrophic lateral sclerosis patients stratified by the D50 progression model

**DOI:** 10.1093/braincomms/fcae389

**Published:** 2024-11-05

**Authors:** Pedram Parnianpour, Robert Steinbach, Isabelle Jana Buchholz, Julian Grosskreutz, Sanjay Kalra

**Affiliations:** Division of Neurology, Faculty of Medicine, University of British Columbia, Vancouver, British Columbia V6T1Z3, Canada; Neuroscience and Mental Health Institute, University of Alberta, Edmonton, Alberta T6G2S2, Canada; Department of Neurology, Jena University Hospital, Jena 07747, Germany; Precision Neurology of Neuromuscular Diseases, University of Lübeck, Lübeck 23538, Germany; Cluster of Excellence of Precision Medicine in Inflammation (PMI), Universities of Lübeck and Kiel, Lübeck 23538, Germany; Precision Neurology of Neuromuscular Diseases, University of Lübeck, Lübeck 23538, Germany; Cluster of Excellence of Precision Medicine in Inflammation (PMI), Universities of Lübeck and Kiel, Lübeck 23538, Germany; Neuroscience and Mental Health Institute, University of Alberta, Edmonton, Alberta T6G2S2, Canada; Division of Neurology, Faculty of Medicine and Dentistry, University of Alberta, Edmonton, Alberta T6G2B7, Canada

**Keywords:** amyotrophic lateral sclerosis, magnetic resonance imaging, texture analysis, d50 disease progression model, disease stratification

## Abstract

Amyotrophic lateral sclerosis, a progressive neurodegenerative disease, presents challenges in predicting individual disease trajectories due to its heterogeneous nature. This study explores the application of texture analysis on T1-weighted MRI in patients with amyotrophic lateral sclerosis, stratified by the D50 disease progression model. The D50 model, which offers a more nuanced representation of disease progression than traditional linear metrics, calculates the sigmoidal curve of functional decline and provides independent quantifications of disease aggressiveness and accumulation. In this research, a representative cohort of 116 patients with amyotrophic lateral sclerosis was studied using the D50 model and texture analysis on MRI images. Texture analysis, a technique used for quantifying voxel intensity patterns in MRI images, was employed to discern alterations in brain tissue associated with amyotrophic lateral sclerosis. This study examined alterations of the texture feature autocorrelation across sub-groups of patients based on disease accumulation, aggressiveness and the first site of onset, as well as in direct regressions with accumulation/aggressiveness. The findings revealed distinct patterns of the texture-derived autocorrelation in grey and white matter, increase in bilateral corticospinal tract, right hippocampus and left temporal pole as well as widespread decrease within motor and extra-motor brain regions, of patients stratified based on their disease accumulation. Autocorrelation alterations in grey and white matter, in clusters within the left cingulate gyrus white matter, brainstem, left cerebellar tonsil grey matter and right inferior fronto-occipital fasciculus, were also negatively associated with disease accumulation in regression analysis. Otherwise, disease aggressiveness correlated with only two small clusters, within the right superior temporal gyrus and right posterior division of the cingulate gyrus white matter. The findings suggest that texture analysis could serve as a potential biomarker for disease stage in amyotrophic lateral sclerosis, with potential for quick assessment based on using T1-weighted images.

## Teaser

Parnianpour *et al*. investigated texture analysis on T1-weighted MRI of patients with amyotrophic lateral sclerosis in relation to the D50 disease progression model. Based on consistent associations between disease accumulation/phase and texture-derived autocorrelation, the latter could thus serve as unimodular neuroimaging biomarker for disease stage.

## Graphical Abstract

**Graphical Abstract:**
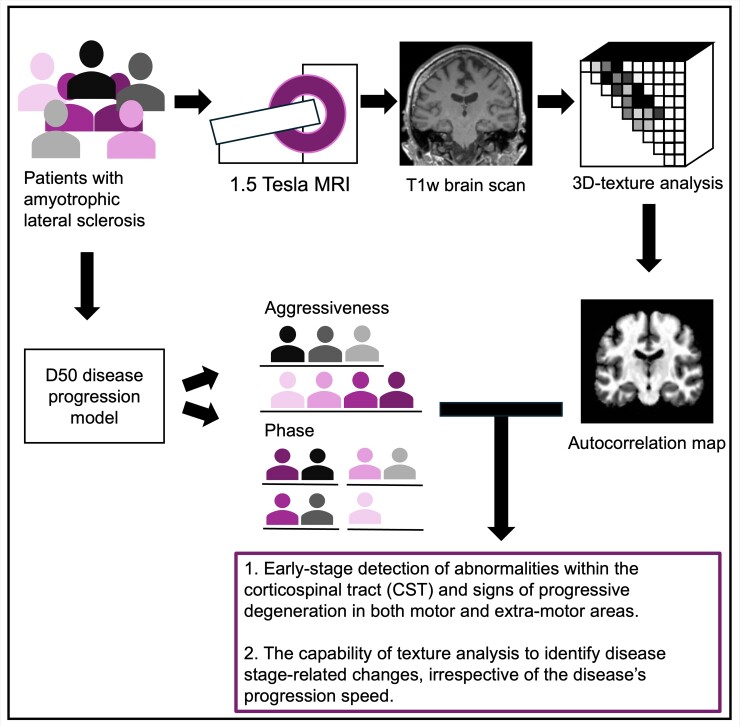

## Introduction

Amyotrophic lateral sclerosis (ALS) constitutes a profoundly heterogeneous and progressive neurodegenerative disorder characterized by its preferential impact on upper and lower motor neurons. The intricate heterogeneity within the ailment is attributed to an array of contributory elements, including (poly-) genetic variant profiles, initial symptoms manifestations, varying disease progression rates, extent of non-motor involvement and the sequential dissemination of pathological processes. Enhanced comprehension of the contribution of each implicated determinant enhances the capacity to anticipate the trajectory of disease advancement.^1^ However, the individual disease course remains only partly predictable and uniquely tailored to each ALS patient. It thus seems paramount to establish a model, which would allow independent and reliable staging of patients regarding disease accumulation and aggression, not only concerning clinical interest but also more personalized therapy approaches and timely implementation of potentially necessary medical aids.

The D50 disease progression model was developed to overcome limitations of traditional clinical metrics, most of all imprecise linear approximations of the temporal decline of function (e.g. the disease progression rate).^2^ The sigmoidal curve of declining motoric functions is calculated based on all available ALS functional rating scale-revised (ALSFRS-R) scores of a given patient and allows the calculation of independent measures of disease aggressiveness as well as disease accumulation. It thus enables unbiased comparisons of patients with vastly differing time courses of the disease in cross-sectional cohorts (so-called pseudo longitudinal approach). The D50 model has already been proven to facilitate robust correlations with (potential) biomarker signals originating from MRI analyses as well as other sources.^3-6^

The first application of D50 in structural MRI analyses used voxel-based morphometry and was able to show sequential worsening and spreading of cortical and subcortical atrophy along with increasing spatial involvement of disease, as revealed in sub-group analyses.^7,8^ Further, more detailed analysis of grey matter measuring cortical thickness and subcortical deep grey matter volume showed robust correlations with disease accumulation, independent of disease aggressiveness.^9^ However, as also suggested from voxel-based morphometry analysis before, disease aggressiveness correlated with the extent of white-matter changes, as assessed via diffusion tensor imaging (DTI).^4^

Texture analysis is an image processing technique aimed at quantifying intricate patterns and inter-dependencies among voxel intensities within an image.^10^ The textural patterns extracted are not detectable by the naked eye on the original image. In this study, a voxel-based grey level co-occurrence matrix on three orthogonal planes in 3D space (VGLCM-TOP-3D) was used to derive texture maps from MR images.^11^ Texture feature, autocorrelation, was calculated based on second-order statistical moments inherent in voxel signal intensities, contingent upon predefined directions and distances.^12^ The application of this method has unveiled deviations in tissue texture along the corticospinal tract of ALS patients.^13-15^ Furthermore, recent findings highlight the method sensitivity to disease progression, as longitudinal assessment exhibited texture modifications within both grey matter and white matter regions of ALS patients’ brains.^16^

This study aimed at using an unbiased approach of parallel assessment of grey and white matter structures via texture analysis and to correlate these with parameters derived from the D50 model. Based on prior results we hypothesized that (i) the texture feature autocorrelation would show grey-matter as well as white matter alterations associated with higher disease accumulation and (ii) white-matter alterations associate with higher disease aggressiveness.

If successful, this would qualify T1-weighted MRI-derived texture analysis to serve as potential unimodular disease stage biomarkers with the advantage of rapid assessment.

## Materials and methods

### Participants and inclusion/exclusion criteria

This study was conducted in accordance with the Declaration of Helsinki and its later amendments. All experiments received prior approval from the local Ethics Committee of the Friedrich Schiller University in Jena (3633-11/12) and all participants signed written informed consent. Patients with ALS were recruited from the centre for Neuromuscular and Motor Neuron Diseases at Jena University Hospital (Jena, Germany).

A diagnostic certainty of at least ‘probable laboratory-supported ALS’ (or higher) needed to be met according to the revised El Escorial criteria,^17^ as assessed by a neurodegenerative specialist. Patients with comorbidities that could affect motor performance were excluded, along with cases of juvenile ALS, primary lateral sclerosis, or manifest dementia. Healthy Controls (HCs) were recruited from the general population and did not have any morbidities affecting cognitive or motor functions, as assessed anamnestically by a physician experienced in neurodegenerative conditions upon inclusion in the study.

### Clinical characterisation with the D50 model

The D50 disease progression model describes the disease state transition as a sigmoidal curve from full health to functional loss. It is calculated by iterative fitting of regularly gathered ALSFRS-R scores for each patient. In summary, the D50 value refers to the estimated number of months from symptom onset until a patient loses half of their motor functions. Based on the observation that 30 represents the median of D50 values in this cohort (see also Table 1) in accordance with other cohorts studied before, patients could thus be subdivided into a low aggressive (LA) sub-group (D50 > 30 months), separately from a high aggressive (HA) sub-group (D50 ≤ 30 months).^18,19^ Normalising the D50-value onto 0.5 yields the relative D50 (rD50), which measures individual disease covered/accumulation, independent of disease aggressiveness. The disease course, as indicated by rD50, can be categorized into different phases: (i) an early semi-stable Phase I (rD50 < 0.25), (ii) an early progressive Phase II (0.25 ≤ rD50 < 0.50) and (iii) late progressive or stable Phases III/IV (rD50 ≥ 0.50). In this study, patients were classified based on their individual rD50 at the time of their MRI scan, placing them either in Phase I (rD50 < 0.25) or Phase II (0.25 ≤ rD50). The number of subjects in Phases III/IV was insufficient for a comprehensive assessment of these stages.

**Table 1:** Demographic and clinical data for patients with ALS

Demographic	ALS*, n* = 116
Age at MRI [years]#	63.08 ± 15.36 (27.25–82.91)
Gender [male/female]$	69/47 (59.5%/40.5%)
**Disease metrics**	
Symptom duration [months]#	15 ± 16 (2–136)
Onset [bulbar/spinal]$	34/82 (29.3%/70.7%)
D50 [months]#	30.23 ± 22.53 (3.51–176.36)
Low aggressiveness (D50 ≥ 30)$	59 (50.9%)
High aggressiveness (D50 < 30)$	57 (49.1%)
Relative D50 (rD50)⊞	0.28 ± 0.13 (0.05–0.70)
Phase I at MRI (rD50 < 0.25)$	49 (42.2%)
Phase II (0.25 ≤ rD50 ≤ 0.50)	64 (55.2%)
Phases III/IV (0.50 ≤ rD50)	3 (2.6%)

Continuous data are summarized for ⊞ as mean ± standard deviation or for # as median ± inter-quartile range (each with the total range in brackets). For $ categorical data, the number of cases is given (with percentages in brackets). Variables that are time point dependent refer to the day of MRI acquisition; others depict constant characterisation of patients’ overall disease course.

### Data acquisition and quality check

MRI scans were obtained using a 1.5 Tesla Siemens Sonata Scanner at Jena University Hospital, employing a FLASH 3D sequence with 192 sagittal slices (repetition time: 15 ms, echo time: 5 ms, flip angle: 30°). The field of view was 240 mm × 256 mm, with a slice thickness of 1 mm and a pixel size of 1 mm × 1 mm, using a standard 4-channel head coil. The T1-weighted images were scanned in an acquisition time of ∼12 min as part of a whole scanning protocol that in total lasted ∼45 min. All T1-weighted images had been acquired in the beginning of the scanning session and acquisition schemes were equal in-between subjects.

All T1-weighted raw images underwent visual inspection for imaging artefacts, including blurring, ringing effects and other contrast-related distortion by an experienced rater (done by PP).

### Image processing

Pre-processing of the T1-weighted 3D images was conducted within CAT12^20^ and SPM12 (version 7487 for MRI/VBM data on MacOS Monterey; Welcome Trust Centre for Neuroimaging; https://fil.ion.ucl.ac.uk/spm/software/spm12/) on MatlabR2021a Surface. To align the final texture maps to the Montreal Neurological Institute (MNI) standard space, deformation fields were calculated using T1-weighted images through the high-dimensional Diffeomorphic Anatomical Registration Through Exponentiated Lie Algebra method.^21^ The native-space T1-weighted images of each participant underwent segmentation to extract grey matter, white matter and CSF. Subsequently, the CSF signal was excluded from further analyses. Lastly, non-uniformity variation correction and global intensity standardisation were conducted using CAT12 default parameters.

Texture analysis was performed on bias-corrected and realigned T1-weighted images in the native space of individual participants. Utilising the VGLCM-TOP-3D method,^11^ three-dimensional orthogonal texture maps were computed using statistical parametric mapping version 8 (SPM8) software, accessible at http://www.fil.ion.ucl.ac.uk/spm/software/spm8. This involved determining a Gray-level co-occurrence matrix (GLCM) for each voxel, taking into account its neighbourhood according to a specified direction and distance. The VGLCM-TOP-3D approach calculates texture features autocorrelation by considering GLCMs in three orthogonal planes. The final reported texture feature value is an average derived from all three planes. During GLCM computation, default parameters were employed, encompassing a neighbourhood radius of 1, distance of 1, quantisation level of 8 and a smoothing kernel of 0. Subsequent to texture map computation, a normalisation process was applied to align the maps with the MNI standard space. This normalisation was executed using the deformation fields that had been computed during the initial pre-processing stage.

Labelling of clusters revealed in the subsequent second level analyses was conducted with the FSLeyes version 0.34.2 in the FSL toolbox (Version 6.0.4).^22^

### Statistical analysis

Within the ALS patient group, sub-group comparisons were made for: (i) Phase I versus Phase II and (ii) low disease aggressiveness versus high aggressiveness. Additionally, regression analysis were conducted using the parameters rD50 and D50. In these sub-group and regression analyses, sex, age, disease onset (bulbar versus spinal) and the congruent parameter to the one being analysed were included as nuisance covariates. For instance, in the comparison of rD50-derived phases, sex, age, disease onset type and D50 were accounted for as covariates. We also compared the phase sub-groups with the group of HCs, using age and sex as nuisance covariates. The significance level was set at *P* < 0.0005 and a cluster extent threshold of 10 voxels was applied.

The SPSS^®^ software programme (IBM^®^, v27.0.0.0) was used to conduct statistical analyses of demographic and clinical data. Non-normal distribution of these variables was examined using the Shapiro–Wilks test. Continuous variables are accordingly expressed as mean with standard deviation; whereas, skewed variables are reported as median with inter-quartile range (categorical variables are given as absolute numbers/percentage). Comparison of group-wise averages was appropriately conducted either with a two-sample *t*-test, a Mann–Whitney U-test, or a χ^2^ test.

## Results

### Demographics

Comparing the group of patients with ALS to HCs, there were no significant differences in the distribution of gender (ALS: 47 females; HCs: 36 females; *P* = 0.102). However, patients were significantly older than HCs (ALS: 63.08 ± 15.36; HCs: 53.87 ± 14.28; *P* < 0.001). Further detailed clinical and demographic data is given in Table 1.

In the direct comparison of ALS sub-groups, patients in Phase II were older than those in Phase I (Phase I: 58.91 ± 17.13; Phase II: 65.67 ± 10.64; *P* = 0.001; for further information see Supplementary Table S1). There was no significant age difference between patients with varying levels of disease aggressiveness (see Supplementary Table S2 for more details). Comparing patients with spinal to those with bulbar onset of symptoms, there were no significant distributions concerning age, D50 or rD50 values. However, patients with bulbar onset of symptoms had a higher proportion of female participants (bulbar-onset: 20/58.8% female; spinal-onset: 27/32.9% female; *P* = 0.01). Sex, age and disease onset were applied as nuisance covariates in all the statistical analyses, to correct for potentially interfering co-variates whilst balancing the level of correction in-between all analyses.

### Texture-derived autocorrelation in relation to disease accumulation

**Figure 1:**
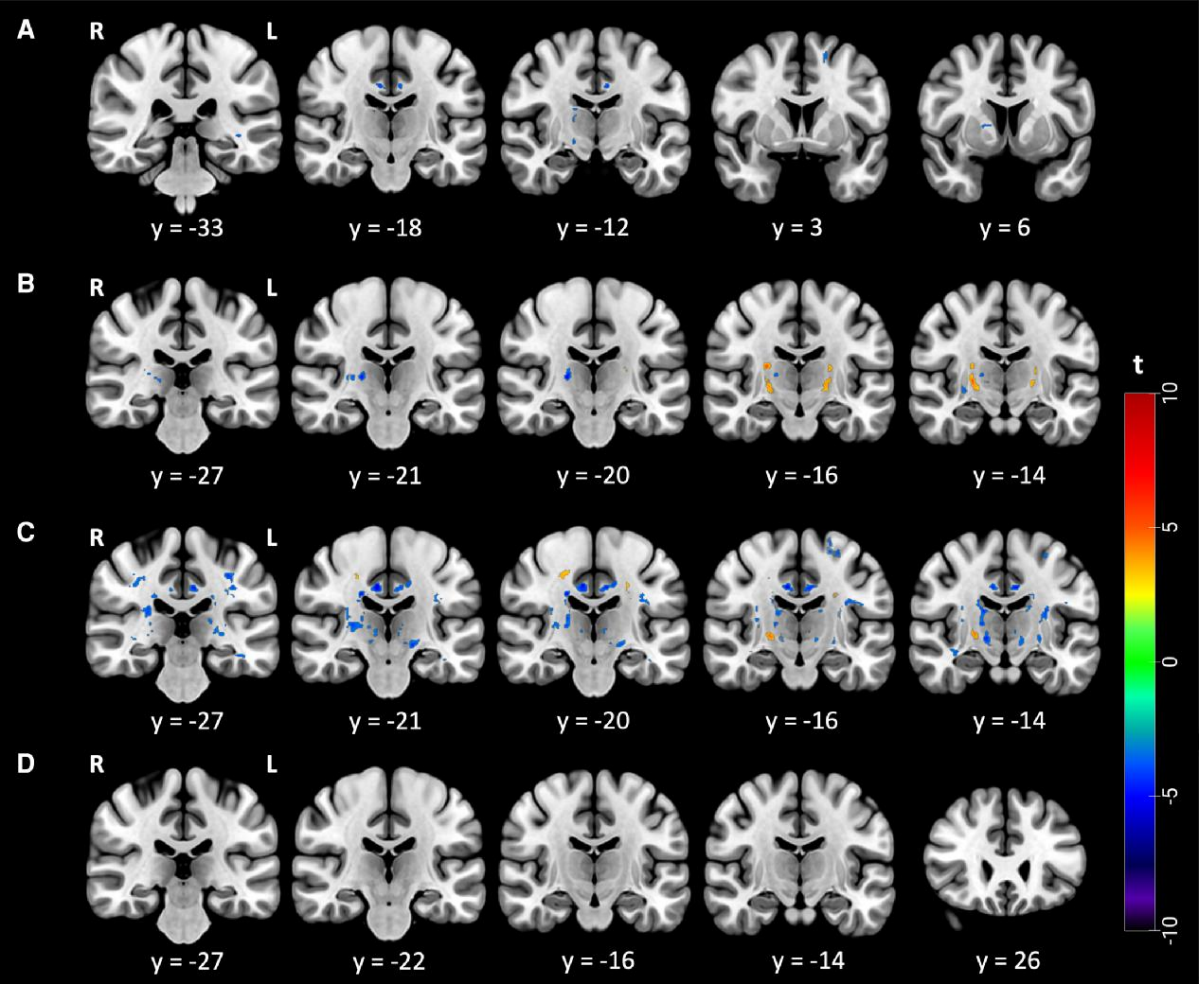
**Group comparison based on the disease accumulation and aggressiveness**. Using a two-sample *t*-test, panel (**A**): Phase 2 > Phase 1, texture differences between Phase 1 (*N* = 49) and Phase 2 (*N* = 64) (*P* < 0.0005, cluster size >10 voxels). (**B**) Phase 1 > HCs, texture differences between Phase 1 and HCs (*N* = 68) (*P* < 0.0005, cluster size >10 voxels). (**C**) Phase 2 > HCs, texture differences between Phase 2 and HCs (*P* < 0.0005, cluster size >10 voxels). (**D**) HA > LA, texture differences between patients with the HA (*N* = 57) and LA (*N* = 59) disease (*P* < 0.0005, cluster size >10 voxels). The colour bar shows the range of *t*-values.

Examining the difference between patients in Phases I and II, distinct clusters were observed within the superior division of the lateral occipital cortex, right thalamus, left cerebellum, bilateral cingulate gyrus, superior frontal gyrus, right cerebral peduncle, retrolenticular part of the left internal capsule, right anterior limb of the internal capsule, left posterior thalamic radiation and left tapetum with a decrease in autocorrelation among patients in Phase II when compared to those in Phase I (Fig. 1A). Notably, there were no increases in autocorrelation in Phase II as compared to Phase I.

In the inter-group contrast, patients in Phase I showed clusters of increased autocorrelation in the lower levels of bilateral corticospinal tract (mostly within the internal capsule) and right grey matter of the hippocampus entorhinal cortex as well as clusters of decreased autocorrelation in the right thalamus, bilateral putamen and left body of the corpus callosum as compared to HCs (Fig. 1B). For the patients in Phase II, increased autocorrelation was observed in bilateral more rostral parts of the corticospinal tract and left temporal pole. Decreased autocorrelation was observed in a widespread pattern and in the bilateral cingulum, right superior thalamic radiation, the right body of the corpus callosum, bilateral arcuate fasciculus, bilateral thalamus, left corticospinal tract, right postcentral gyrus, right anterior corona radiata, bilateral external capsule, right superior longitudinal fasciculus, left caudate, right posterior limb of the internal capsule, right fornix, bilateral retro-lenticular part of the internal capsule, bilateral precentral gyrus, left middle longitudinal fasciculus, left grey matter primary somatosensory cortex BA3a, bilateral inferior longitudinal fasciculus, left anterior thalamic radiation, bilateral optic radiation, left inferior fronto-occipital fasciculus and left anterior limb of the internal capsule (Fig. 1C).

**Figure 2:**
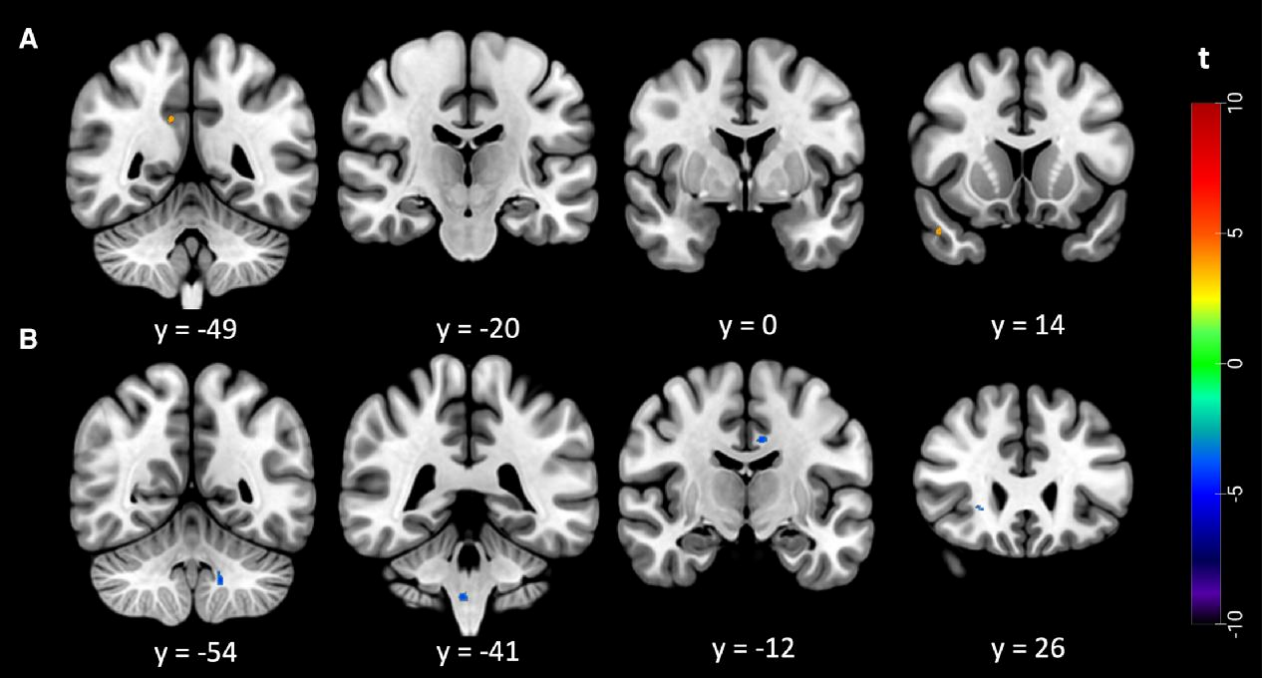
**Voxel-wise regression analysis of the patients’ autocorrelation texture maps.** (*N* = 116) with panel (**A**): Regression with D50 (*P* < 0.0005, cluster size >10 voxels) and (**B**) Regression with rD50 (*P* < 0.0005, cluster size >10 voxels). The colour bar shows the range of *t*-values.

Voxel-wise regression analysis revealed a negative correlation between rD50 and autocorrelation in clusters within the left cingulate gyrus white matter, brainstem, left cerebellar tonsil grey matter and right inferior fronto-occipital fasciculus (Fig. 2A). There was no positive correlation with rD50.

### Texture-derived autocorrelation in relation to disease aggressiveness

A comparison between patients with high aggressiveness and those with low aggressiveness revealed no significant differences in the established threshold (Fig. 1D).

Voxel-wise regression analysis revealed a positive association of autocorrelation with the parameter D50 in clusters within the right superior temporal gyrus white matter and right posterior division of the cingulate gyrus white matter (Fig. 2B). There were no negative correlations with D50.

### Comparison between spinal and bulbar

**Figure 3:**
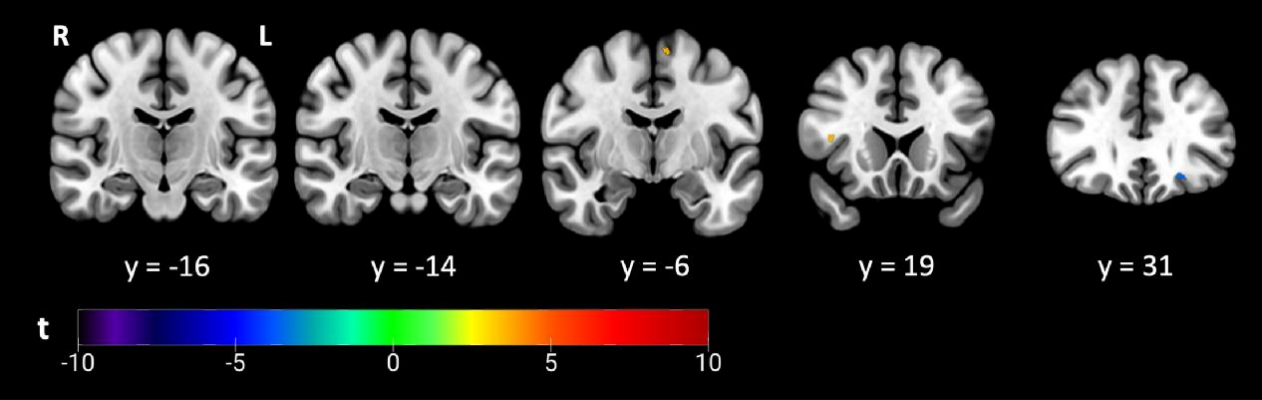
**Group comparison based on the disease site of onset.** Using a two-sample *t*-test, Spinal > bulbar, texture differences between patients with spinal (*N* = 82) and bulbar (*N* = 34) onset (*P* < 0.0005, cluster size >10 voxels). The colour bar shows the range of *t*-values.

Considering the site of onset as a categorical variable, patients exhibiting bulbar onset displayed lower autocorrelation values in both the right superior longitudinal fasciculus and the left superior thalamic radiation. Conversely, a noticeable increase was observed within the left frontal orbital cortex in patients with bulbar onset (Fig. 3).

## Discussion

The objective of this study was to assess cerebral alterations through texture analysis in relation to disease characteristics quantified by the D50 disease progression model. The rD50 values were used to categorize patients into the initial two phases of the disease. Here, patients in Phase II exhibited decreased autocorrelation values across various regions within both grey and white matter, in comparison to those in Phase I. A negative correlation was observed between four distinct clusters and rD50 in voxel wise regression analysis.

The D50 values were used to classify patients with high and low levels of disease aggressiveness, where no notable differences emerged. However, the regression analysis unveiled a positive correlation between two clusters within white matter and D50.

Additionally, patients were further categorized based on their onset site, drawing comparisons between spinal onset and bulbar cases. Individuals with a bulbar onset demonstrated decreased autocorrelation within the internal capsule and white matter tracts in the frontal lobes and increased autocorrelation in the grey matter of the frontal lobe.

The comparative analysis between Phases I and II patients identified regions implicated in the core pathology of ALS, namely motor neuron degeneration, as well as alterations in non-motor areas. These changes are indicative of the progressive and widespread nature of ALS, and they are often overshadowed by the more prominent motor impairments, especially when the comparison is made with HCs.

The study observed a decline in autocorrelation within motor-related regions with increasing disease accumulation, specifically within the right cerebral peduncle, the retrolenticular part of the left internal capsule, the left cerebellar hemisphere (area IX) and the right anterior limb of the internal capsule. The cerebral peduncle plays a vital role in connecting the cerebrum with the brainstem and is integral to motor control. This is supported by evidence indicating a reduction of fractional anisotropy in DTI analysis, with changes beginning from the right corticospinal tract and extending into the right cerebral peduncle.^23^ The internal capsule, which is composed of myelinated fibres that communicate between the cerebral cortex, the brainstem and the spinal cord, has been identified as a primarily affected region in ALS, particularly when analysing the left posterior limb using DTI.^24^ Previous texture analyses of T1-weighted MRIs have consistently highlighted changes in the bilateral internal capsule in ALS,^16,25^ suggesting that neuropathological progression beyond the posterior limb is a feature of the disease’s evolution. Moreover, recent research has pointed to the cerebellum as a previously overlooked element in ALS pathology.^26^ Considering the cerebellum functions in motor coordination and precision, its involvement in the later stages of ALS aligns with the expanding scope of recognized disease impacts. In addition, recent study also suggests the possibility of spinal imaging indicating predictiveness of functional decline,^27^ which could potentially further improve accuracy in combination with neuroimaging at brain level and implementation of the D50 model to stratify patients.

In areas not primarily associated with motor function, the study found reduced autocorrelation in the superior division of the lateral occipital cortex, the right thalamus, the bilateral cingulate gyrus, the left posterior thalamic radiation, the superior frontal gyrus and the left tapetum. This finding is consistent with previous texture analysis of T1-weighted images in the Canadian ALS Neuroimaging Consortium (CALSNIC) cohort.^25^ These regions, while not central to the primary pathology of ALS, have shown abnormalities in ALS patients in previous research, suggesting that they may become implicated as the disease progresses. Zhang *et al*. noted a decrease in cortical complexity in the occipital cortex of ALS patients, a measure that reflects cortical folding patterns.^28^ Longitudinal studies using voxel-based morphometry and volumetric analysis have demonstrated changes in the thalamus.^29,30^ Agosta *et al*. identified grey matter changes in the cingulate gyrus,^31^ with neuropathological studies corroborating a reduction in neuronal density in the prefrontal cortex^32^ as well as decreased structural connectivity of sensorimotor areas.^33^ Furthermore, changes in the volume of the superior frontal grey matter and white matter have been linked with cognitive and speech deficits in ALS patients.^34^ These findings indicate again that the scope of ALS extends beyond the motor system, affecting a range of cerebral regions as the disease evolves. Future studies should evaluate the presence of genetic variants and its implications on the neuroimaging changes observed. For example, carriers of pathologic C9orf72 hexanucleotide expansions were formerly described to have a higher degree of extramotor involvement, emphasized for the thalamus or frontal regions.^35,36^

Most important, the different analyses revealed congruent results of declining autocorrelation in association with advancing disease accumulation as quantified by the D50 disease progression model. This could be shown in negative regression analysis with the parameter rD50, as well as in sub-group-comparisons of patients in rD50-derived disease phases. Notably, the inverse contrasts did not reveal any suprathreshold clusters, thus indicating that autocorrelation is constantly declining whilst the disease progresses. It is noteworthy that these congruent results could be revealed despite accounting for multiple co-variates in the contrasts, including age that due to its direct inter-correlation with the variable rD50 diminishes the observable disease-accumulation related neuroimaging changes. This is in line with former analyses applying the D50 model on T1-weighted volumetry^7^ or cortical thickness.^9^ Briefly, advantages of the D50 model are that the parameters are calculated in a way minimising rater-variability and selection bias and that biomarker-signals can be correlated with the disease accumulation (rD50) independent of disease aggressiveness (corrected for D50).

However, in inter-group comparisons with HCs, there were observations of increased autocorrelation mainly in bilateral corticospinal tract, emphasising rostral parts for those patients in the more advanced disease Phase II at the time of MRI scanning. Such signals in this major motor-tract have been observed before in the CALSNIC cohort on a case-control level,^16^ and notably, this study found that in a real longitudinal setting increases of autocorrelation in the left internal capsule were mostly evident for a slow progressing ALS sub-group. This could implicate, that changes in the corticospinal tract at the level of the internal capsule are a matter of the earlier disease course (as in Phase I or prior) whilst those patients with a faster disease progression had already progressed to more advanced disease stage at the time of MRI scanning. However, this emphasizes the importance to consider characteristics of ALS patient cohort as a whole as measures of disease accumulation/state and progression-speed/aggressiveness are usually highly inter-correlated in observational/cross-sectional ALS cohorts (see also Supplementary Tables S1 and S2). This phenomenon has been identified and discussed as ‘sampling shift’ before.^5^

The results of the study presented here, although MRI scans acquired at 1.5 Tesla, show convincing similarities with those observed in the independent CALSNIC cohort at 3 Tesla, thus highlighting the ability of T1-weighted MRI texture analysis to non-invasively mirror the progression of ALS pathology in the brain.^14,16,25^

Texture analysis of T1-weighted images did not show any variations among patient sub-groups when categorized by disease aggressiveness levels within the established threshold. However, positive correlations between two small clusters in juxtacortical white matter and D50 values were observed in voxel wise regression analysis, indicating declined autocorrelation in these areas associated with higher disease aggressiveness. This is in line with former study where disease aggressiveness was described to show a high correlation with white matter tract changes as examined in DTI analysis.^4^ The clusters were revealed next to the right-hemispheric superior temporal gyrus and cingulate gyrus, areas that have been described in ALS neuroimaging studies before to be associated with a higher degree of cognitive dysfunction.^37,38^ Future studies including thorough neurocognitive profiling are therefore needed to see if sub-clinical cognitive impairment contributes to these changes, also typically associated with faster progression in ALS.

After all, texture analysis in the examined feature of autocorrelation seems as a tendency more prone to changes associated with disease accumulation than disease aggressiveness. This can be concluded from the negative direct sub-group comparisons of patients with different disease aggressiveness levels, whilst the comparison of patients in Phase I with those in Phase II showed congruent results as with direct regression analysis with the parameter rD50. Although a former texture analysis study by Ishaque *et al*. observed differences between slow and fast progressors compared to HCs, as stratified based on the linear rate of disease progression,^16^ the most recent study showed this pattern of changes is similar in patients stratified by both King’s staging system and disease progression rate.^25^ This enhances the possibility of having differences in disease accumulation among sub-groups of fast and slow progressors and points again towards the importance to consider possibly inter-correlated covariates. Current findings may suggest that the methodology employed is more sensitive to the cumulative effects of the disease rather than to the rate of its progression.

Patients with bulbar onset showed lower autocorrelation in the right superior longitudinal fasciculus III and left superior thalamic radiation, while an increase was observed in the left frontal orbital cortex compared to those with spinal onset. These observations are consistent with a previous study that utilized voxel-based morphometry and tract-based spatial statistics, which confirmed significant alterations in patients with bulbar onset.^8^ Such findings may point again to more pronounced frontotemporal changes and internal capsule involvement in patients with bulbar onset ALS.

This study is not without limitations. This a monocentric study that requires reproduction of the neuroimaging results in independent cohorts. The D50-model is a framework that allows to perform pseudo-longitudinal analyses of the cohort. However, verification through real longitudinal data are needed but naturally will depend on large, well-designed multi-centre studies.^39,40^ Furthermore, genetic testing was not available for all patients, which is why we could not characterize them based on genetic profiles. Similarly, the patients were not neuropsychologically tested and therefore subclinical cognitive deficits and/or behavioural impairment are possible. Future studies with more advanced acquisition schemes, e.g. using a higher MRI field strength such as 3 Tesla are also needed to assess if this would produce different neuroimaging results in relation to the clinical model. Although texture analysis yields promising results that align closely with the clinical pathology of ALS, *ex vivo* studies are needed to uncover the neuropathological basis of the microstructural changes in tissue texture. In addition, a complete workup of possible unrecognized comorbidities in the HC group (e.g. including physical examination and cognitive testing) was not available and we therefore cannot rule out conditions possibly interfering with the neuroimaging analyses.

## Conclusions

In this study, we conducted texture analysis on T1-weighted images in a representative cohort of patients with ALS. By application of the D50 disease progression model, we were able to examine relations between the neuroimaging feature autocorrelation with disease accumulation independent of disease aggressiveness and vice versa. As hypothesized, alterations in autocorrelation in white as well as grey matter structures were associated with disease accumulation. On the other hand, disease aggressiveness correlated with only two small clusters of white matter changes in voxel-wise regression analysis. However, there were no associations between autocorrelation changes in grey matter and aggressiveness, thus also confirming our second a-priori hypothesis. Therefore, it can be concluded that the neuroimaging method used here might serve as a unimodular tool to assess disease accumulation in ALS.

## Supplementary Material

fcae389_Supplementary_Data

## Data Availability

The data that support the findings of this study are available on request from the corresponding author.
