# Supplementary material for: T1-weighted MRI texture analysis in amyotrophic lateral sclerosis patients stratified by the D50 progression model
Source: Brain Commun. 2024 Nov 5;6(6):fcae389. doi: 10.1093/braincomms/fcae389 (PMC11562117; doi:10.1093/braincomms/fcae389)
Supplement: fcae389_Supplementary_Data [file fcae389_supplementary_data.docx]

# **SUPPLEMENTARY MATERIAL**

# **Supplementary Table 1:**

| **ALS (*n* = 113)** | **Phase I** | **Phase II** | ***p*** |
| --- | --- | --- | --- |
| *Demographics* |  |  |  |
| n | 49 | 64 |  |
|  | (43.4%) | (56.6%) |  |
| age at MRI [years] # | 58.91 ± 17.13 | 65.67 ± 10.64 | *0.001* |
|  | (31.83 - 82.91) | (27.25 - 82.83) |  |
| gender [male/female] ⊚ | 35/14 | 34/30 | *0.054* |
|  | 71.4%/ 28.6% | 53.1%/ 46.9% |  |
| *Traditional Disease Metrics* |  |  |  |
| symptom duration [months] # | 9 ± 9 | 19 ± 13 | *< 0.001* |
|  | (4 - 73) | (2-136) |  |
| onset [bulbar/spinal] ⊚ | 16/33 | 16/48 | *0.405* |
|  | 32.7%/ 67.3% | 35.0%/ 75.0% |  |
| *D50 disease progression model parameters* |  |  |  |
| D50 [months] # | 33.05 ± 21.62 | 29.06 ± 22.45 | *0.067* |
|  | (10.59 - 176.36) | (3.51 - 168.22) |  |
| relative D50 [rD50] ⊞ | 0.15 ± 0.06 | 0.35 ± 0.07 | *< 0.001* |
|  | (0.05 - 0.249) | (0.25 - 0.49) |  |
| # non-parametric Mann-Whitney-U-Test |  |  |  |
| ⊞ parametric t-statistic |  |  |  |
| ⊚ chi-squared test |  |  |  |
| *Abbreviations*: *D50* estimated time in months for an individual to lose 50% of functionality; *rD50* (relative D50) individual disease covered; *Note*: Continuous data are summarized for ⊞ as mean ± SD and for # as median ± interquartile range (each with the total range in brackets). For ⊚ categorial data the number of cases and percentages are given. Variables that are time-point dependent refer to the day of MRI-acquisition; others depict constant characterization of patients’ overall disease course. | | | |

# **Supplementary Table 2:**

| **ALS (*n* = 116)** | **Low Aggressiveness** | **High Aggressiveness** | ***p*** |
| --- | --- | --- | --- |
| *Demographics* |  |  |  |
| n | 59 | 57 |  |
|  | 50.9% | 49.1% |  |
| age at MRI [years] # | 62.16 ± 16.42 | 64.66 ± 16.13 | *0.564* |
|  | (31.83 – 78.75) | (27.25 - 82.91) |  |
| gender [male/female] ⊚ | 35/24 | 34/23 | *0.971* |
|  |  |  |  |
| Traditional Disease Metrics |  |  |  |
| symptom duration [months] # | 23 ± 17 | 11 ± 9 | *<0.001* |
|  | (4 - 136) | (2 - 29) |  |
| onset [bulbar/spinal] ⊚ | 14/45 | 20/37 | *0.179* |
|  | 23.7%/ 76.3% | 35.1%/ 64.9% |  |
| D50 disease progression model parameters |  |  |  |
| D50 [months] # | 41.8 ± 22.36 | 20.51 ± 11.81 | *<0.001* |
|  | (30.04 - 176.36) | (3.51 - 29.88) |  |
| relative D50 [rD50] ⊞ | 0.25 ± 0.13 | 0.3 ± 0.13 | *0.076* |
|  | (0.05 - 0.49) | (0.06 - 0.7) |  |
| # non-parametric Mann-Whitney-U-Test |  |  |  |
| ⊞ parametric t-statistic |  |  |  |
| ⊚ chi-squared test |  |  |  |
| *Abbreviations*: *D50* estimated time in months for an individual to lose 50% of functionality; *rD50* (relative D50) individual disease covered; *Note*: Continuous data are summarized for ⊞ as mean ± SD and for # as median ± interquartile range (each with the total range in brackets). For ⊚ categorial data the number of cases and percentages are given. Variables that are time-point dependent refer to the day of MRI-acquisition; others depict constant characterization of patients’ overall disease course. | | | |
